# Supplementary material for: Fe-Sn nanocrystalline films for flexible magnetic sensors with high thermal stability
Source: Sci Rep. 2019 Mar 1;9:3282. doi: 10.1038/s41598-019-39817-8 (PMC6397158; doi:10.1038/s41598-019-39817-8)
Supplement: Supplementary file 1 — SUPPLEMENTARY INFO [file 41598_2019_39817_MOESM1_ESM.pdf]

Supplementary information for
Fe-Sn nanocrystalline films for flexible magnetic sensors with high thermal stability

Y. Satake[1], K. Fujiwara[1*], J. Shiogai[1], T. Seki[1,2], and A. Tsukazaki[1,2]

[1]*Institute for Materials Research, Tohoku University, Sendai 980-8577, Japan*

[2]*Center for Spintronics Research Network (CSRN), Tohoku University, Sendai 980-8577, Japan*

*Author to whom correspondence should be addressed.
Electronic mail: kfujiwara@imr.tohoku.ac.jp

1, Structural analysis of polycrystalline Fe-Sn films

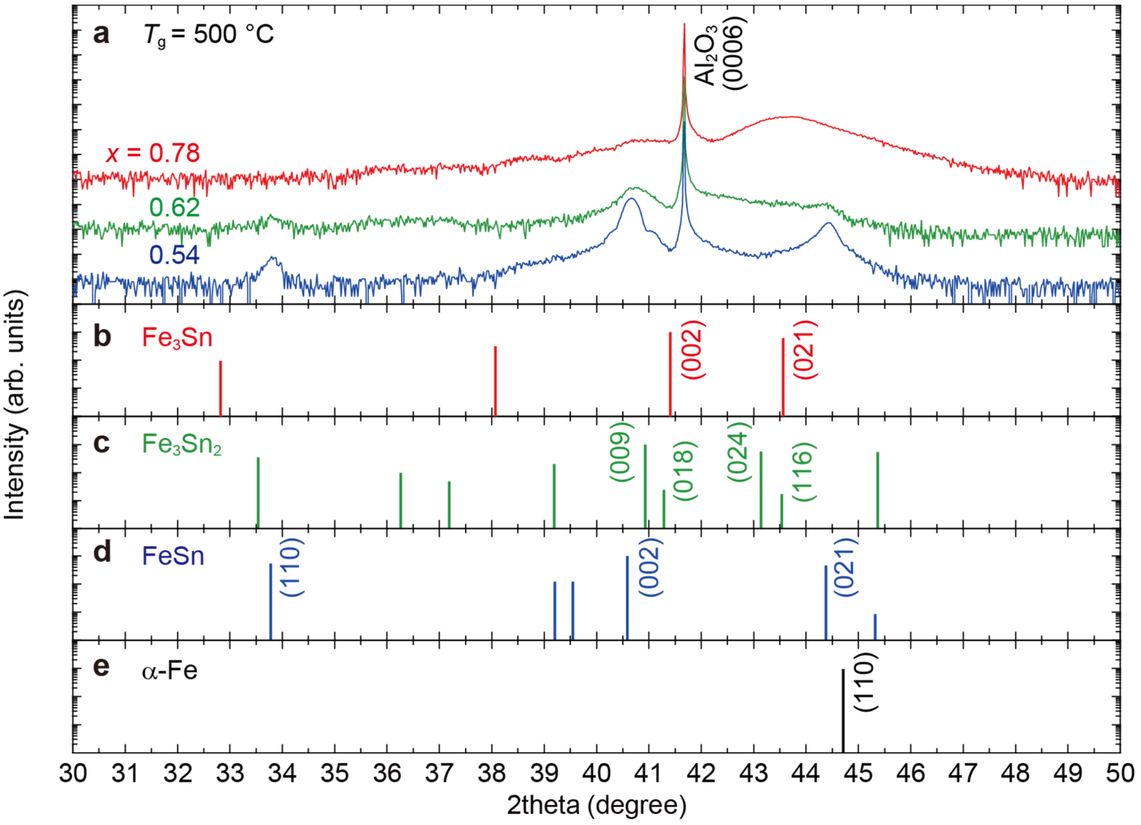

Figure S1 | XRD peak assignment of Fe-Sn films deposited at 500 °C. a, XRD patterns for Fe_xSn_{1-x} films with x = 0.54, 0.62 and 0.78, fabricated on sapphire (0001) substrates at 500 °C. **b**-**e,** Reference data are taken from the Inorganic Crystal Structure Database (ICSD) for Fe_3Sn (**b,** ICSD 24569), Fe_3Sn_2 (**c,** ICSD 71), FeSn (**d,** ICSD 103634), and α-Fe (**e,** ICSD 53451).

In Fig. S1, XRD patterns of Fe_xSn_{1-x} films fabricated by high-temperature sputtering are compared with possible references. For x = 0.54, peaks centered at 2θ = 33.8°, 40.6° and 44.4° are indexed as FeSn (110), (002) and (021), respectively, indicating that the film is dominated by antiferromagnetic FeSn. For x = 0.62, additional broad

reflections emerge around 41°and 43°, which can be assigned to Fe_3Sn_2 (009) and (024). Therefore, ferromagnetic Fe_3Sn_2 likely exists in a form of small domains embedded within the antiferromagnetic FeSn matrix. According to preceding work on annealing-induced crystallization of Fe-Sn, the broad peak around 43.5° observed for x = 0.77 should be of Fe_3Sn (021) (Supplementary ref S1), though the possibility of Sn-rich α-Fe is not completely excluded only with this measurement.

2, Transmission electron microscopy (TEM) analysis for nanocrystalline $Fe_{0.60}Sn_{0.40}$.

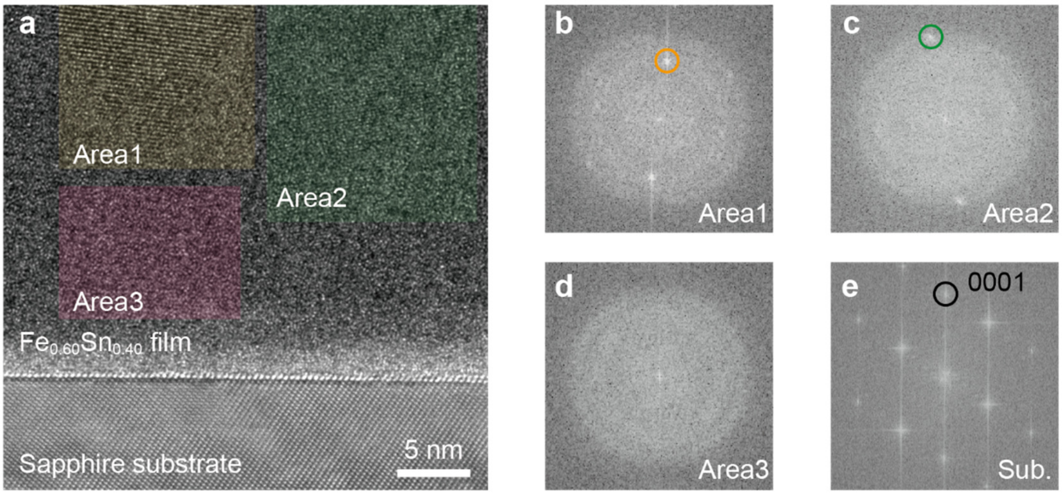

Figure S2 | Microstructure observation of room-temperature sputtered $Fe_{0.60}Sn_{0.40}$. **a,** Cross-sectional high-resolution TEM image of a $Fe_{0.60}Sn_{0.40}$ film grown on sapphire (0001) substrate at room temperature. **b–e,** Fast Fourier transformed diffractograms collected from the areas emphasized by overlaid colours in Fig. S2a: (**b**) from Area1, (**c**) from Area2, (**d**) from Area3 and (**e**) from the substrate.

Figure S2a shows a low-magnification TEM image of the data shown in Fig. 1f. Although the presence of crystalized domains is not detected in the room-temperature sputtered $Fe_{0.60}Sn_{0.40}$ films by XRD (Fig. 1e), the formation of layered nanocrystals is clearly visible in this image. To characterize the nanocrystalline structures, we performed fast Fourier transform analysis for some regions denoted as Area1, 2 and 3 (Fig. S2b-d). Using the (0006) spot of sapphire substrate (Fig. S2e) as the reference in *k*-space, *d*-spacing values are estimated to be 2.98 Å from Area1 (yellow circles in Fig. S2b) and 2.08 Å from Area2 (green circles in Fig. S2c). The former can be indexed as Fe_3Sn_2 (006)

(d = 3.30 Å, ICSD 71) and also β-Sn (200) (d = 2.91 Å, ICSD 106072), and the latter as FeSn (002) (d = 2.24 Å, ICSD 103634), Fe_3Sn_2 (009) (d = 2.21 Å, ICSD 71), Fe_3Sn (002) (d = 2.18 Å, ICSD 24569) and α-Fe (110) (d = 2.03 Å, ICSD 53451). In Area3, no specific spots are recognized (Fig. S2a). It is difficult to identify exact phases only with these rough estimates of d-spacings as well as limited information about crystal symmetry. However, β-Sn and α-Fe should be less likely because the characteristic layered stacking is clearly resolved. In addition, based on the comparative study on the antiferromagnetic FeSn-dominant polycrystalline film (Fig. 2c,d), if a large portion of FeSn is included in the nanocrystalline film, the large AHE should have not been observed. It is thus reasonable to consider that the crystalline phases are ferromagnetic Fe_3Sn_2 and/or Fe_3Sn kagome metals. The dome-like composition dependence of AHE characteristics shown in Fig. 2e, at least, excludes that one phase predominates over the other one; their fractions gradually increase with approaching the stoichiometric compositions. Therefore, we conclude that the film with x = 0.60, corresponding to Fe_3Sn_2, contains Fe_3Sn_2-like domains in the amorphous matrix.

3, In-plane magnetization

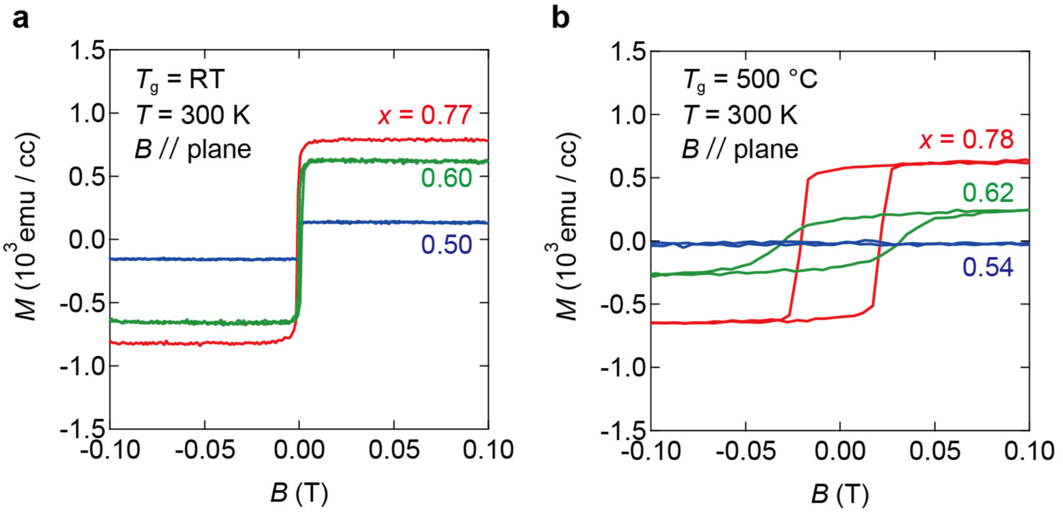

Figure S3 | In-plane magnetization curves for Fe-Sn alloy films. a, Magnetization M measured for nanocrystalline Fe_xSn_{1-x} films on sapphire (0001) at 300 K as a function of an in-plane magnetic field B. **b,** Results for polycrystalline Fe_xSn_{1-x} films on sapphire (0001).

Figure S3a illustrates the small saturation magnetic field in the in-plane measurement configuration. This contrasts to the out-of-plane data (Fig. 2b) where much larger B is needed to saturate M. By high-temperature sputtering, the hysteresis of M versus B curves develops (Fig. S3b). These results mean that the magnetization easy axis exists in the film plane.

4, *T* dependence of saturation magnetization

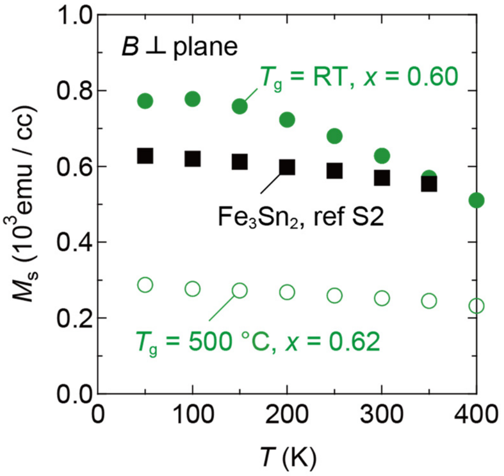

Figure S4 | *T* dependence of saturation magnetization. Saturation magnetization M_s measured in an out-of-plane B. M_s of nanocrystalline $Fe_{0.60}Sn_{0.40}$ films on sapphire (0001) (filled green circles), polycrystalline $Fe_{0.62}Sn_{0.38}$ films on sapphire (0001) (open green circles) and Fe_3Sn_2 bulk (black squares, calculated from data in Supplementary ref S2) are plotted.

M_s of Fe_3Sn_2 bulk in literature (Supplementary ref S2) weakly increases as T is decreased. The Currie temperature T_C of Fe_3Sn_2 is 657 K. A similar dependence is observed for the polycrystalline $Fe_{0.62}Sn_{0.38}$ film grown at 500 °C (open green circles) while M_s is only half the bulk value. The M_s reduction is attributed to the effective decrease of ferromagnetic components due to the formation of antiferromagnetic FeSn. M_s in the nanocrystalline $Fe_{0.60}Sn_{0.40}$ film grown at room temperature (filled green circles) is comparably large to the bulk M_s. The slight difference in their T dependences might imply additional minor M contributions from unidentified ferromagnetic phases in the nanocrystalline film.

5, Bending experiments on PEN sheets

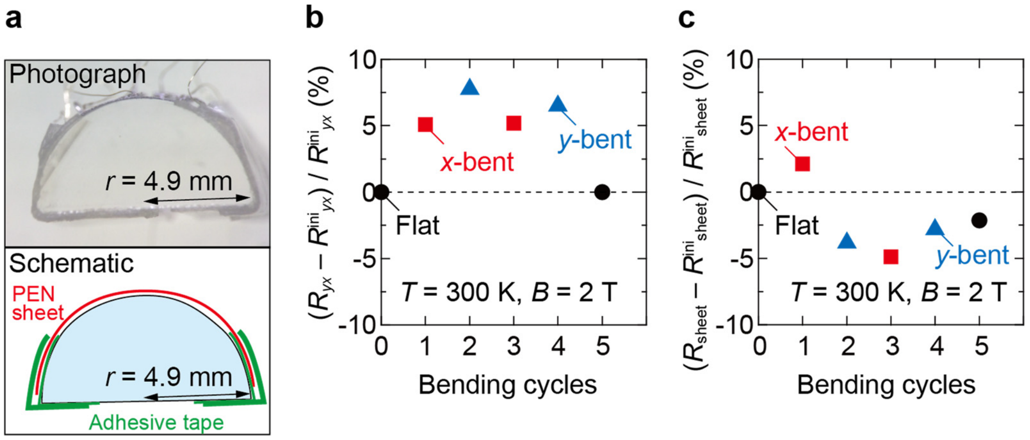

Figure S5 | Bending effect on transport properties. a, Cross-sectional photograph (top panel) and corresponding schematic (lower panel) of a 4-nm-thick nanocrystalline $Fe_{0.60}Sn_{0.40}$ device on a PEN sheet under bending experiments. The device is attached to the surface of a 4.9-mm-radius semicircular jig with an adhesive tape. **b,c,** Hall and sheet resistance change ratios against bending cycles, defined as $R_{yx} = (R_{yx} - R^{ini}{}_{yx}) / R^{ini}{}_{yx}$ ($R^{ini}{}_{yx}$: Hall resistance before bending) and $(R_{sheet} - R^{ini}{}_{sheet}) / R^{ini}{}_{sheet}$ ($R^{ini}{}_{sheet}$: longitudinal resistance before bending). Black circles, red squares and blue triangles represent flat, *x*-bent and *y*-bent configurations displayed in Fig. 4g, respectively.

A typical photograph of device bent with a semicircular jig is presented in Fig. S5a (also see Fig. 4g). By repeatedly mounting and removing the device in different configurations, the reversible operation was confirmed (Fig. S5b,c).

6, Universal relationship of σ_{xy} and σ_{xx}

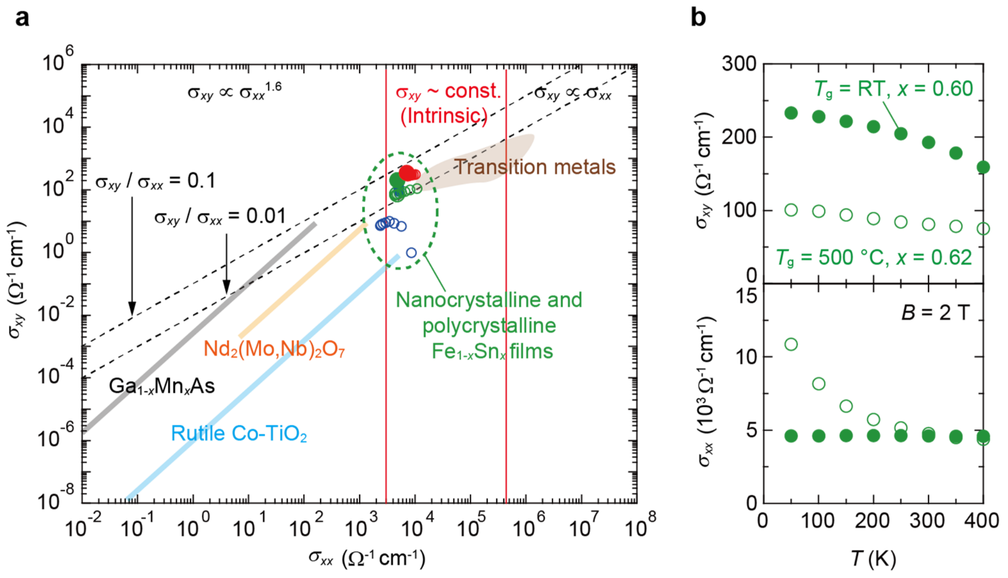

Figure S6 | σ_{xy} versus σ_{xx} relations in Fe_xSn_{1-x} films. a, σ_{xy} is plotted against σ_{xx} for nanocrystalline and polycrystalline Fe_xSn_{1-x} on sapphire (0001), and also some representative materials exhibiting large AHEs. Color codes (red , green and blue) are the same to those in Fig. 2. The panel is made based on Supplementary ref S3. **b,** σ_{xy} (upper panel) and σ_{xx} (lower panel) as a function of T.

The σ_{xy} versus σ_{xx} plot, shown in Fig. S6a, provides a useful guide to discussing AHE origins (Supplementary ref S3). According to the widely accepted theory, σ_{xx} of ~ 3 × 10^3 Ω^{-1}cm^{-1} is the critical border that separates the poorly conductive region and intrinsic region. Our Fe_xSn_{1-x} films are located in the intrinsic region. In parallel to this, the polycrystalline $Fe_{0.62}Sn_{0.38}$ film grown at 500 °C (open green circles) exhibit virtually constant σ_{xy} while σ_{xx} depends on T (Fig. S6b), in agreement with the intrinsic AHE origin.

Although σ_{xy} slightly varies with T in the nanocrystalline film, this is reasonably ascribed to the T dependence of M shown in Fig. S4.

Supplementary References

1. Haftek, E., Tan, M. & Barnard, J. A. Crystal structure and magnetic properties of nanophase Fe-Sn alloy thin films. *Nanophases and Nanocrystalline structure*; San Diego; CA, 1992; Mar, 0, 105–120
2. Ye, L. *et al.* Massive Dirac fermions in a ferromagnetic kagome metal. *Nature* **555,** 638–642 (2018).
3. Onoda, S., Sugimoto, N. & Nagaosa, N. Quantum transport theory of anomalous electric, thermoelectric, and thermal Hall effects in ferromagnets. *Phys. Rev. B* **77,** 165103 (2008).
